# Sclerosing Angiomatoid Nodular Transformation of the Spleen: A Diagnostic Conundrum

**DOI:** 10.5334/jbsr.2689

**Published:** 2022-04-05

**Authors:** Sander Van den Eede, Nick Van de Voorde, Filip Vanhoenacker, Bart Op de Beeck

**Affiliations:** 1 University of Antwerp, BE; 2 Antwerp University Hospital and Antwerp University, Edegem, Belgium, BE; 3 AZ Sint-Maarten, Mechelen, Belgium, BE; 4 AZ Sint-Maarten and University (Hospital) Antwerp/Ghent, BE

**Keywords:** Sclerosing angiomatoid nodular transformation, Splenic lesions, Multimodality imaging, Image guided biopsy, Splenectomy

## Abstract

A splenic lesion often represents a diagnostic challenge due to relative scarcity and the broad differential diagnosis. Sclerosing Angiomatoid Nodular Transformation (SANT) of the spleen is usually encountered only incidentally. Although benign, patients with SANT often receive splenectomy, due to its rarity, diagnostic uncertainty and sometimes intimidating imaging morphology and size. Imaging features on computed tomography, magnetic resonance and positron emission tomography have a high diagnostic value for SANT and help differentiate this entity from other splenic lesions. When the imaging parameters are matched with core needle biopsy tissue analysis, further watchful waiting can be recommended in order to avoid splenectomy.

## Introduction

Sclerosing angiomatoid nodular transformation (SANT) is a rare benign, proliferative vascular lesion affecting the spleen with approximately 170 cases described in scientific literature. SANT is first described as a distinct pathology by Martel et al. in 2004 [1]. Its etiology is still unclear. Patients are mostly middle-aged adults, with a slight female preponderance [2]. SANT is often asymptomatic and therefore mostly encountered incidentally. These lesions may be large at presentation and may mimic malignancy, often leading to splenectomy. The diagnosis of SANT is almost always made only on histopathologic examination after splenectomy. Considering the risk of splenectomy, the purpose of this manuscript is to review the imaging features of SANT, in addition to our own case, in order to examine whether imaging may suggest the diagnosis. Furthermore, the value and safety of core needle biopsy is reviewed.

## Methodology

A systematic search was performed in the database Medline, using PubMed as search engine. The following search terms were used: (Sclerosing angiomatoid nodular transformation) OR (Splenic lesions) OR (splenic tumor) OR (Splenectomy). After the initial search we selected publications by title, subsequently by abstract. If the article did not contain enough imaging information to implement in this review, it was not included in the analysis. Ultimately 33 articles were considered eligible in this review in addition to our own case, containing 58 cases describing the imaging features of SANT, summarized in ***Table 1***. The time window of the reviewed literature ranges from 2004 until 2019 [1,15].

**Table 1:** Characteristics of SANT based on a review of literature.


Age	Average: 44 years (range: 17–80)

Gender	M: 45% (26/58)/F: 55% (32/58)

Clinical findings	Asymptomatic: 61.1% (22/.6) Left upper quadrant pain: 13.5% (5/36) Nonspecific abdominal complaints: 19.4 % (7/36) Right upper quadrant pain: 2.7% (1/36) Fatigue: 2.7%(1/36)

Morphology	Lobulated: 58.6% (17/29) Smooth borders: 31.0 % (9/29) Central calcification: 37.9% (11/29) Radiating/central scar: 31,0% (9/29)

Computed Tomography	Hypodense: 93.8% (45/48) Isodense: 6.3% (3/48) Enhancement pattern: – Peripheral enhancement in the arterial phase with progressive filling in: 93% (27/29) – Spoke-wheel pattern: 44% (13/29)

Magnetic Resonance Imaging	T1-WI – Hypointense: 51.6%(17/33) – Isointense: 39.3% (13/33) – Hyperintense: 9.1% (3/33) T2-WI – Hypointense: 65.6% (21/32) – Isointense: 12.5%(4/32) – Hyperintense: 21.9% (7/32) Enhancement pattern – Peripheral enhancement in the arterial phase with progressive filling in: 79% (18/23) – Spoke-wheel pattern: 48% (11/23) – Central scar: 26% (6/23)

Ultrasound	Hypoechoic: 78.6% (11/14) Isoechoic: 14.2% (2/14) Hyperechoic: 7.1% (1/14) Spoke-wheel enhancement pattern on CEUS: 100% (4/4) Persistent peripheral enhancement on CEUS: 75% (3/4)

FDG-PET	Lowavidity: 42.9 % (6/14) Heterogeneous avidity: 42.9 % (6/14) High avidity: 14.3% (2/14) Mean SUVmax: 2.8 MBq/g(range: 2.0–4.7 MBq/g)


Abbreviations: M = Male; F = Female; n = number; CEUS = Contrast Enhanced Ultrasound; FDG-PET = Fluoro-deoxyglucose-Positron Emission Tomography; SUVmax = Maximal Standardized Uptake Value.

## Characteristics

SANT is an infrequent benign vascular lesion and often an incidental finding. There is a slight female predominance. The mean age in the study population was 44 years, correlating well with other, not included reviews [2,3]. SANT is almost exclusively described in the spleen, except for one reported at the adrenal gland [4]. At diagnosis these lesions may be large with a mean diameter of 49 to 175 mm [3].

The etiology of SANT remains unknown. In the literature there are several hypotheses. For instance, Martel et al. suggest a non-neoplastic stromal proliferative process in splenic red pulp tissue with accompanied nodular followed by vascular transformation [1]. Weinreb et al. reports a possible association with Epstein-Barr Virus and Kuo et al. considered an IgG4-related sclerosis in the pathogenesis of SANT [5,6].

SANT can be distinguished from other vascular lesions of the spleen based on its distinct histologic findings and immunohistologic features. The most characteristic microscopic findings of SANT are multiple angiomatoid nodules separated by collagenous bundles [1]. Immunohistochemically, these nodules are composed of three types of blood vessels: the cord capillary type, CD31+/CD34+/CD8–; the sinusoid type, CD31+/CD34–/CD8+ and the small vein type, CD31+/CD34–/CD8– (1).

No specific symptoms are associated with SANT. Up to 62,3% of the cases, patients were asymptomatic. A minority of 13.5% of patients report non-specific abdominal pain or discomfort in the left upper quadrant. Only one report describes a patient with life-threatening intra-abdominal hemorrhage and severe abdominal distention, requiring urgent splenectomy [7].

## Imaging findings

**Figure 1:**
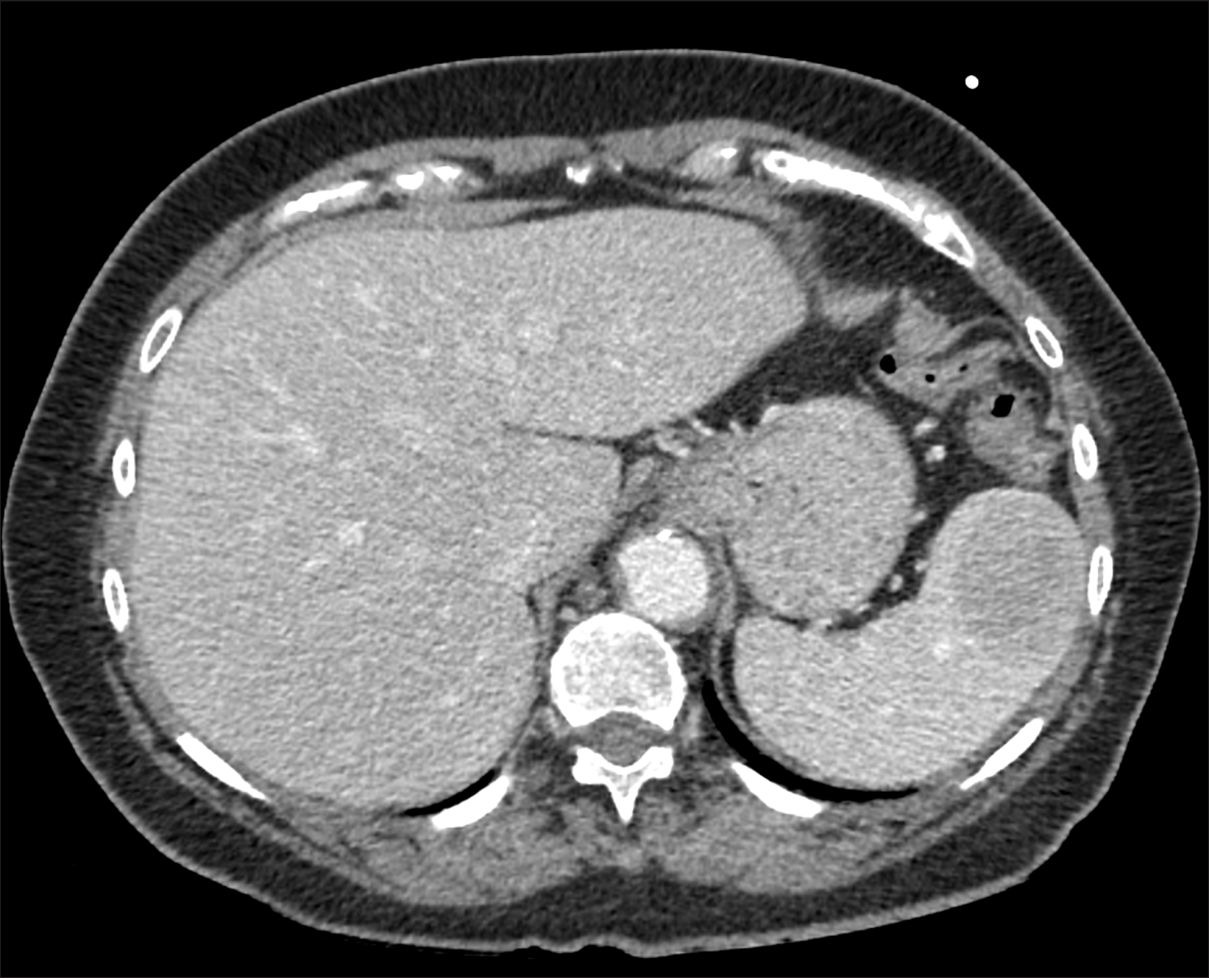
Axial Computed Tomography in the porto-venous phase depicts a sharply delineated lesion anteriorly in the spleen with relative hypo-enhancement compared to the surrounding splenic parenchyma.

**Figure 2:**
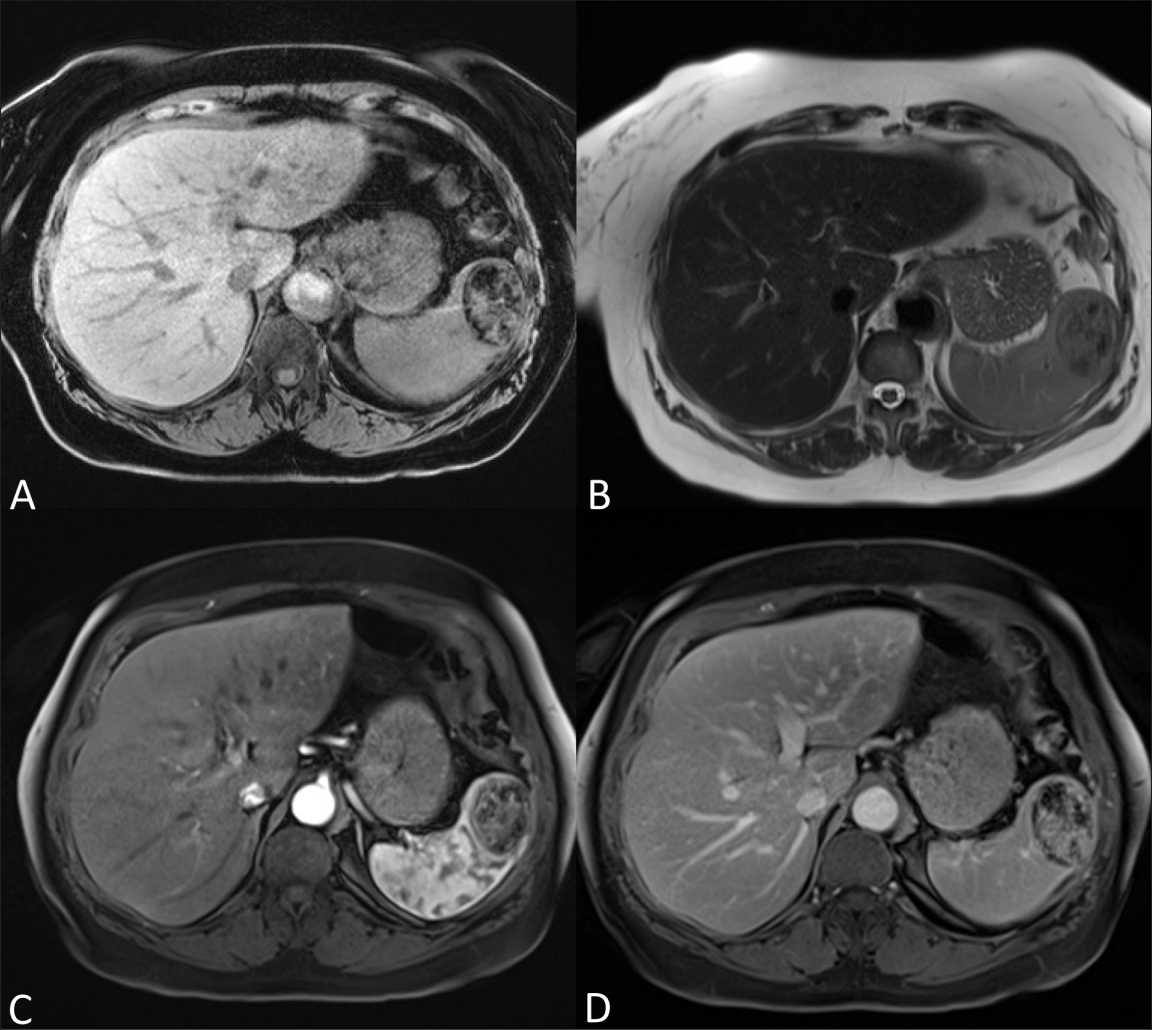
(A) Unenhanced axial fat suppressed T1-weighted imaging shows the lesion is sharply delineated with abundant hypointense foci. (B) Axial T2-weighted imaging shows a sharply delineated heterogeneous mass with both iso- and hypointense components. (C–D) Axial fat suppressed T1-weighted imaging after injection of gadolinium contrast shows a relative heterogeneous and weak enhancement in the arterial phase progressively filling in at the delayed phase, although remaining heterogeneous. No central scar or spoke-wheel enhancement pattern was present.

Imaging findings of histologically proven SANT are summarized in ***Table 1***. Across the 33 studies the patients either underwent ultrasound, computed tomography (CT), magnetic resonance imaging (MRI) and/or fluoro-deoxyglucose-positron emission tomography (FDG-PET) investigations. Most articles contain imaging information on CT, where SANT presents as a hypodense solitary splenic mass in 94% (45/48) of cases and 93% (27/29) report peripheral enhancement and progressive filling in on the delayed phase (***Fig. 1***). Some articles describe the enhancement as a ‘spoke-wheel’ pattern, corresponding to a central, stellate fibrous stroma with fibrous septa separating angiomatoid nodules [3,8,9,10,11,12,13,14,15]. Morphologically SANT is lobulated in 59% (17/29) of cases and 31% (9/29) describe SANT as a lesion with smooth borders. A considerable number of CT cases (38%) show central calcifications, however almost exclusively reported by one author [16]. MRI characteristics of SANT are reported in 37 of the reviewed cases. On T1-WI 52% (17/32) of lesions are hypointense and 39% isointense compared to normal splenic parenchyma (***Fig. 2A***). On T2-WI, 66% (21/33) of articles report abundantly low signal intensities (***Fig. 2B***). This is most likely due to hemosiderin deposition [17]. The stellate radiating bands extending toward the center are reportedly T2-hypointense due to their fibrous composition [8]. The periphery of SANT is hyperintense to the surrounding tissue on T2-WI in only 22% of cases (7/32). The enhancement pattern on multiphase MRI is similar to CT, consisting of nodular enhancement of the periphery in the early arterial phase with a progressive centripetal filling through radiating septa in the delayed phase (***Fig. 2C–D***) [8,9,10,15,16,17,18,19,20,21]. A central scar may be seen on T1- and T2-WI and was present in 26% of cases [8,10,17,22,23].

The sonographic characteristics of SANT was only mentioned in 14 articles. The most common presentation is a solitary heterogeneously hypoechoic mass [9,11,24,25,26,27]. Sporadic case reports have examined the possible utility of contrast-enhanced ultrasonography (CEUS) in the examination of SANT [11,25,26,27]. Gutzeit et al. describes the enhancement of SANT on CEUS as a spoke-wheel pattern similar to the findings on multi-phase CT and MRI [25].

**Figure 3:**
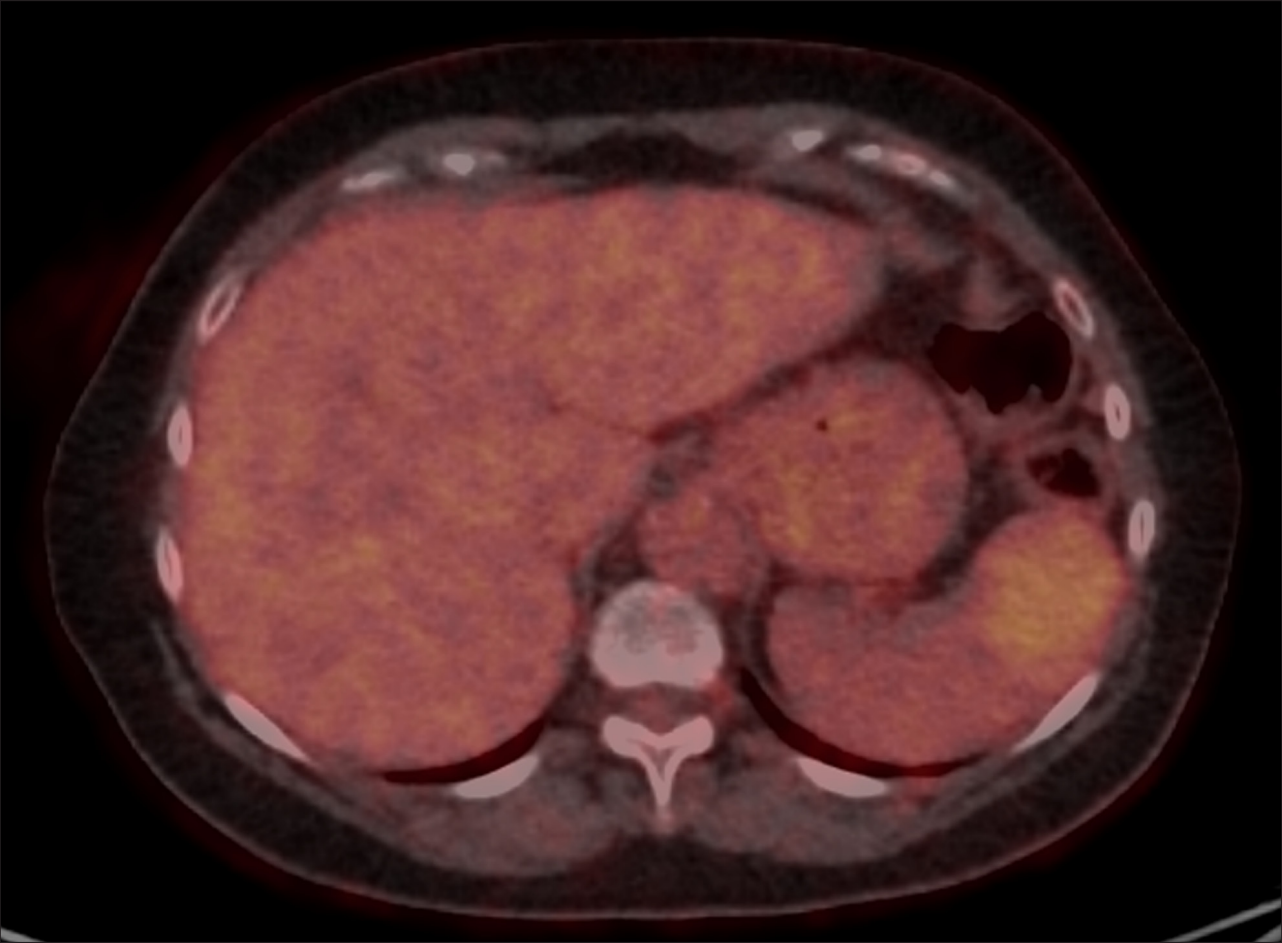
Axial Fluoro-deoxyglucose-Positron Emission Tomography shows a moderate uptake in the splenic lesion. Extra-splenic lesions were absent.

SANT cannot be distinguished from other benign splenic tumors based on FDG-PET findings alone, but it can be a helpful tool to differentiate between certain malignant tumors such as lymphoma, metastases and angiosarcoma [28,29,30]. In addition, FDG-PET can exclude distant disease, typically absent in SANT (***Fig. 3***). Fourteen authors performed FDG-PET in the work-up. Only a moderate uptake was noted with a median maximal standardized uptake value (SUVmax) of 2.8 MBq/g (range: 2.0–4.7 MBq/g), as opposed to malignant tumors with higher average values such as lymphoma that has a median SUVmax of 6.9MBq/g [28].

## Differential diagnosis

Primary splenic masses can be categorized into those of lymphoid and non-lymphoid origin [31]. Lymphoid tumors are the most common neoplasms of the spleen. Non-lymphoid tumors are infrequent and are mostly of vascular origin, such as hemangiomas [32]. Although rare, SANT should be considered in the differential diagnosis of splenic lesions. The main differential diagnosis of SANT includes lymphoma, angiosarcoma, littoral cell angioma, hamartoma, hemangioma, metastasis and extramedullary hematopoiesis [33].

*Lymphoma* is the most common malignancy involving the spleen. Typical findings, not present in SANT, include splenomegaly, intra-abdominal lymphadenopathy and high FDG avidity [34]. On MRI these lesions are generally T1- and T2-hypointense with typical diffusion restriction on diffusion-weighted images (DWI) due to high cellularity [35].

*Hemangioma*, or more correctly termed venous malformation, is the most common benign splenic lesion and may resemble SANT. Prominent hyperintensity on T2-WI and multiplicity may be helpful in the differentiating this lesion from SANT [36].

Although richly vascularized, the spleen is an uncommon site for *metastatic* disease explained by the parenchyma’s natural ability to resist metastases [37]. The most frequently reported primary tumors to metastasize to the spleen are cutaneous melanoma, lung cancer and breast cancer, usually in an advanced disease stadium (33). FDG-PET is useful for differentiating splenic metastases from SANT, showing high FDG uptake like the primary tumor [30].

*Angiosarcoma* is an aggressive tumor arising from splenic endothelial cells with very poor prognosis, as distant metastases are usually present at the time of initial diagnosis [38]. Splenic angiosarcoma presents as diffuse infiltrative mass. Important discriminators between angiosarcoma and SANT are the washout of contrast medium on the delayed phase for angiosarcoma as opposed to the progressive centripetal ‘spoke wheel’ enhancement for SANT and the abundantly high uptake at FDG-PET [29].

*Littoral cell angioma* (LCA) is a rare benign vascular tumor that may mimic SANT [36]. However, LCA can be differentiated from SANT due to its typical multiple hypodense nodules of varying size. In addition, LCA is more likely to be symptomatic, for up to 50% of patients present with anemia and thrombocytopenia [33,36].

Splenic *hamartoma* is an uncommon non-neoplastic lesion composed of an abnormal mixture of white and red pulp. One key feature of a splenic hamartoma is its faint heterogeneous enhancement on immediate post-contrast images, with relatively uniform and intense enhancement on delayed images [33,39]. In addition, hamartomas show high FDG uptake compared to low to moderate uptake by SANT [40].

*Extramedullary hematopoiesis* (EMH) is defined as the production of blood cells outside of the bone marrow. The most common causes of EMH are myelofibrosis, diffuse osseous metastatic disease replacing the bone marrow, leukemia, sickle cell disease, and thalassemia [41]. EMH of the spleen most commonly manifests as splenomegaly without a discrete mass. Very rarely, EMH presents as a focal splenic mass. SANT can be differentiated from extramedullary hematopoiesis by its typical high intensity on T2-WI and hypermetabolism displaying high FDG avidity on PET-CT [35,41].

## Imaging guided biopsy

In 98% of reviewed cases the patient with SANT underwent splenectomy. The main reason for surgical resection is diagnostic uncertainty on imaging studies. However, it should be noted that the spleen is an lymphoid organ, with an important role for general immunity and splenectomy is not an innocent intervention [42]. Intra- or postoperative hemorrhage, thromboembolic complications and overwhelming post-splenectomy infection cause considerable morbidity and mortality [43]. Lehne et al. found an associated mortality rate of 2.9% and a morbidity rate of 37%, with a higher frequency of sepsis and pneumonia even two years after splenectomy [44].

Moreover, in-depth splenic immune function studies suggesting unnecessary splenectomy for benign lesions leads to the loss of an important immunologic organ are widely accepted [45].

Of the reviewed cases, only Gutzeit et al. and our own case obtained the final diagnosis of SANT through image guided core needle biopsy (CNB) [25].

US or CT can both be used for image guidance and the choice is often related to the experience of the interventional radiologist. The advantages of US guided biopsy are the real-time needle visualization, the shorter time needed to reach the target, low cost and no radiation exposure for patient or radiologist. Color doppler sonography aids the radiologist in avoiding any large vessels and plotting a safe trajectory to the lesion. Usually three biopsies are performed, taken from the center and periphery of the lesion to assure enough tissue samples to acquire an adequate pathological diagnosis. Possible disadvantages include impaired visualization of deep lesions of the spleen caused by interposition of intestinal gases, lung parenchyma and overlying ribs. CT guided biopsy of the spleen has several benefits over the ultrasound-guided procedure: the ability to visualize all the abdominal organs and viscera; less risk of bowel transgression and better characterization of deep-seated lesions, collections and retroperitoneal structures [46,47,48].

A common misconception amongst clinicians might be the possibility of a significant complication associated with splenic biopsy, due to the high vascular nature of the spleen. However a meta-analysis found the major complication rate was 1.3% for all splenic biopsies for fine needle aspiration cytology (FNAC) and CNB and to 1.9% for CNB alone, when biopsy needles of 18-gauge or smaller are used [49]. The complication rate rises significantly (4.2%) when biopsies performed with 14-gauge needles are included. This major complication rate is comparable to those reported for CNB of the kidney (0.7%) and liver (0.5%), suggesting that the inherent risk of CNB of the spleen is not substantially greater in experienced hands [50]. The major complication of concern is hemorrhage. Other complications include pneumothorax, pleural effusion and colonic injury.

Image-guided percutaneous biopsy of the spleen performs well and yields high overall diagnostic accuracy. For all biopsies, sensitivity was 87% and specificity was 96.4% [49]. There are sporadic reports of false positive results after ultrasound guided biopsy [51]. Unquestionably, splenectomy remains the reference standard and upholds the highest diagnostic accuracy when differentiating splenic lesions. However, given the lower complication rate and far less invasive nature of biopsy, the trade-off is acceptable [49].

Absolute contraindications to splenic biopsy are similar to those of any other solid abdominal organ biopsy: significant coagulopathy; severe cardiopulmonary compromise; pregnancy; hemodynamic instability; lack of a safe biopsy pathway, and an uncooperative patient [52].

## Conclusion

The imaging findings of SANT on ultrasound and CT are rather unspecific. The diagnosis of SANT can be suggested on MRI when encountering a lobulated lesion with smooth margins, which is iso- to hypointense on T1-WI and abundantly low on T2-WI compared to splenic tissue, shows peripheral arterial enhancement with delayed filling in or ‘spoke-wheel’ enhancement pattern and only moderate/heterogeneous uptake on FDG-PET. These findings combined with the absence of malignant characteristics such as wash-out, infiltrative growth and adenopathies, make the diagnosis of SANT very likely. When these imaging parameters are matched with core needle biopsy tissue analysis, further watchful waiting can be recommended in order to avoid unnecessary splenectomy. CNB in experienced hands is safer than splenectomy, yields diagnostic material in the majority of cases and should be the next diagnostic step after imaging.

## Main Points

- Typical imaging features of SANT are hypointense signal on T1-WI and T2-WI with peripheral enhancement in the arterial phase with a progressive centripetal filling in the delayed phase, displaying a characteristic spoke-wheel-like pattern in almost half of cases.
- Important discriminators between SANT and malignant splenic lesions are the absence of washout, moderate/heterogenous uptake on FDG-PET, infiltrative growth and adenopathies.
- Core needle biopsy shows high diagnostic accuracy, is safer than splenectomy and should be the diagnostic intervention of choice for solid splenic lesions.

## Additonal Files

The additonal files for this article can be found as follows:

10.5334/jbsr.2689.s1 Patient characteristics and imaging features of Sclerosing Angiomatoid nodular transformation. This table is a summary of all the data acquired of SANT since this entity was first described in 2004. We divided the table in study name, age of patient, sex, clinical features, Computed tomography features, Magnetic resonance imaging features, Positron emission tomography features, Ultrasound features and treatment.
