# Supplementary material for: Sclerosing Angiomatoid Nodular Transformation of the Spleen: A Diagnostic Conundrum
Source: J Belg Soc Radiol. 2022 Apr 5;106(1):12. doi: 10.5334/jbsr.2689 (PMC8992766; doi:10.5334/jbsr.2689)
Supplement: Patient characteristics and imaging features of Sclerosing Angiomatoid nodular transformation. — This table is a summary of all the data acquired of SANT since this entity was first described in 2004. We divided the table in study name, age of patient, sex, clinical features, Computed tomography features, Magnetic resonance imaging features, Positron emission tomography features, Ultrasound features and treatment. [file jbsr-106-1-2689-s1.pdf]

Study	Age	Sex	Clinical	CT features	MRI features	PET features	US features	Treatment
Agrawal et al.(54)	35	M	LUQ pain	Isodense<br>CE: heterogeneous				Splenectomy
Atas et al.(10)	33	M	LUQ pain		T1WI: Isointense<br>T2WI: Hyperintense<br>CE: heterogeneous contrast uptake during the late phase			Laparoscopic splenectomy
Bamboat et al.(10)	17	M	Intermittent abdominal pain	Hypodense lesion with a smooth border	T2WI: Hypointense rim with a focus of hyperintensity toward the center (spoke wheel)	Low avidity		Laparoscopic splenectomy
Budzynski et al.(55)	23	F	Vague abdominal pain	Well-circumscribed highly vascularised lesion				Laparoscopic partial splenectomy
Cipolla et al. (56)	66	F	Epigastric pain	Hypodense lesion	T1WI: Iso-hypointense<br>T2WI: Hypointense with a centrally scattered hyperintense signal			Splenectomy
Demicri et al.(20)	43	M	IF	2 hypodense lesions	T1WI: Hyperintense<br>T2WI: Hypointense		Hypoechoic	Laparoscopic splenectomy
Eusebio et al.(21)	62	F	LUQ pain		T1WI: isointense<br>T2WI: hypointense with hyperintense septae			Splenectomy
Feng et al. (34)	44	F				Heterogeneous avidity (SUV max 2,8)		
Gaeta et al.(12)	54	M	IF	Hypodense mass<br>CE: Early phase shows rim and nodular enhancement of the periphery of the lesion with a few radiating septa, late phase shows heterogenous enhancement with some central areas of low attenuation	T1WI: Heterogeneous hypointense<br>T2WI: Heterogeneous hypointense<br>DWI: Isointense with hypointense areas<br>CE: Peripheral and globular enhancement with progressive centripetal filling	Low avidity (SUVmax 3,3)	Hypoechoic	Laparascopic hemisplenectomy
Gooch et al.(22)	21	F	Fatigue		T1WI: peripheral isointense and central hypointense<br>T2WI: peripheral isointene and central hypointense<br>CE: Early phase shows nodular enhancement of the periphery with a lack of central filling in the delayed phase		Hyperechoic	Laparoscopic splenectomy
Gutzeit et al.(31)	77	M	IF	Hypodense mass with central calcification			Isoechoic with a hypoechoic rim<br>CE: Hypoenhancement in the delayed phase	Core needle biopsy
Immamura et al.(13)	37	F	IF	Hypodense mass<br>CE: Early phase shows enhancement of	T1WI: Hypointense<br>T2WI: Heterogeneous hyperintense	Low avidity (SUVmax 3,7)	Hypoechoic	Laparoscopic splenectomy

				the periphery of the lesion with a few faintly visible septae penetrating the center and progressively enhancement toward its center on the delayed phase	DWI: Hypointense<br>CE: Early peripheral enhancement with progressive centripetal filling, with a persistent non-enhancing central hypointense focus on the delayed phase			
Kakisaka et al.(23)	36	M	IF	Hypodense mass<br>CE: peripheral globular discontinuous enhancement with a gradual centripetal fill-in pattern	T1WI: Hypointense<br>T2WI: Hyperintense			Laparoscopic splenectomy
Karaosmanoglu et al.(11)	44	M	IF	Hypodense mass with lobulated contours	T1WI: Hyperintense<br>T2WI: Hyperintense with central hypointense areas<br>CE: Progressive enhancement from periphery to center of lesion (spoke wheel pattern)			Splenectomy
Kim et al.(24)	39	M	IF		T1WI: Heterogeneous hypointense<br>T2WI: Heterogeneous hypointense	Heterogeneous avidity (SUVmax 2,8)	Heterogeneous hypoechoic	
Kim et al.(24)	40	F	IF				Heterogeneous hypoechoic	
Kim et al.(24)	48	F	Flank pain	Homogeneous hypodense	T1WI: Isointense<br>T2WI: Heterogeneous hypointense			
Kim et al.(24)	43	F	IF	Homogeneous hypodense	T1WI: Heterogeneous hyperintense<br>T2WI: Heterogeneous hypointense		Heterogeneous hypoechoic	
Kim et al.(24)	39	M	IF	Heterogeneous hypodense	T1WI: Heterogeneous hypointense<br>T2WI: Heterogeneous hypointense	Heterogeneous avidity (SUVmax 2,7)		
Kim et al.(24)	50	M	IF	Heterogeneous hypodense	T1WI: Heterogeneous hypointense<br>T2WI: Heterogeneous hypointense	Heterogeneous avidity (SUVmax 2,0)		
Koreishi(57)	58	F	IF			Heterogeneous avidity (SUVmax 4,7)		Splenectomy
Lee et al.(58)	58	M	IF	Hypodense mass		High avidity		Open splenectomy
Lee et al.(14)	38	F	Upper abdominal discomfort	Hypodense mass<br>CE: Peripheral enhancement with progressive centripetal filling			Hypoechoic<br>CE: spoke wheel pattern	Splenectomy
Lewis et al.(25)	35	F		Hypodense mass with lobulated borders	CE: Homogeneous hyperenhancement in the delayed phase			Splenectomy

Lewis et al. (25)	68	F		Hypodense mass with smooth border	CE: Heterogeneous hyperenhancement in the portal phase			Splenectomy
Lewis et al.(25)	31	F		Hypodense with lobulated borders	T1WI: Heterogeneous isointense<br>T2WI: Hypointense<br>CE: Heterogeneous weak enhancement in the early phase and heterogeneous isoenhancement in the delayed phase			Splenectomy
Lewis et al.(25)	37	M		Hypodense mass with lobulated borders	CE: Heterogeneous weak enhancement in the early phase and heterogeneous isoenhancement in the delayed phase			Splenectomy
Lewis et al.(25)	44	M		Hypodense mass with lobulated borders	T1WI: Heterogeneous isointense<br>T2WI: Hypointense<br>CE: Heterogeneous weak enhancement in the early phase and heterogeneous isoenhancement in the delayed phase			Splenectomy
Lewis et al.(25)	27	F		Hypodense mass with lobulated borders	T1WI: Isointense and homogeneous<br>T2WI: Hypointense and homogeneous<br>CE: Heterogeneous weak enhancement in the early phase and homogeneous isoenhancement in the delayed phase			Splenectomy
Lewis et al.(25)	35	F		Hypodense mass with lobulated borders	CE: Heterogeneous weak enhancement in the early phase and homogeneous isoenhancement in the delayed phase			Splenectomy
Lewis et al.(25)	27	F		Hypodense mass with lobulated borders	T1WI: Isointense and homogeneous<br>T2WI: Hypointense and heterogeneous<br>CE: Heterogeneous weak enhancement in the early phase and heterogeneous hyperenhancement in the delayed phase			Splenectomy
Lewis et al.(25)	67	M		Hypodense mass with smooth border	CE: Heterogeneous weak enhancement in the early phase			Splenectomy
Lim et al.(26)	39	M	IF	Hypodense mass with smooth border	T1WI: Isointense<br>T2WI: Hypointense<br>CE: Progressively peripheral enhancement extending centrally in the delayed phase		Hypoechoic	Splenectomy
Ma et al.				Hypodense with central calcification and lobulated border<br>CE: Spoke wheel pattern				Splenectomy
Ma et al.				Hypodense with central calcification and lobulated border				Splenectomy
Ma et al.				Hypodense with central calcification and lobulated border				Splenectomy
Ma et al.				Hypodense with central calcification and lobulated border				Splenectomy

				CE: Spoke wheel pattern				
Ma et al.				Hypodense with central calcification and lobulated border				Splenectomy
Ma et al.				Hypodense with central calcification and lobulated border				Splenectomy
Ma et al.				Hypodense with central calcification and lobulated border<br>CE: Spoke wheel pattern				Splenectomy
Ma et al.				Isodense with central calcification and lobulated border				Splenectomy
Ma et al.				Hypodense with central calcification and lobulated border	T1WI: Hypointense<br>T2WI: Hypointense<br>CE: Spoke wheel pattern			Splenectomy
Ma et al.				Hypodense with central calcification and smooth border	T1WI: Hypointense<br>T2WI: Hypointense<br>CE: Spoke wheel pattern			Splenectomy
Ma et al.					T1WI: Hypointense<br>T2WI: Hypointense<br>CE: Spoke wheel pattern			Splenectomy
Ma et al.					T1WI: Hypointense<br>T2WI: Hypointense<br>CE: Spoke wheel pattern			Splenectomy
Matsubara et al.(27)	64	F	IF	Hypodense mass with smooth border	T1WI: Hypointense<br>T2WI: Hypointense	Low avidity (SUVmax 2,8)		Laparoscopic splenectomy
Menozzi et al.(32)	40	F	Dyspepsia				Isoechoic mass<br>CE: Early phase: spoke-wheel pattern.<br>Delayed phase: slightly hypoechoic	Splenectomy
Nagai et al.(15)	33	M	IF	Hypodense mass<br>CE: Delayed enhancement centripetally,	T1WI: Hypointense<br>T2WI: Heterogeneous hyperintense			Laparoscopic splenectomy

				with a wheel-like appearance				
Nomura et al.(5)	41	F	IF	Hypodense solid mass<br>CE: Hypoenhancment in early and portal phase, isoenhancement in delayed phase	T1WI: Isointense<br>T2WI: Isointense	Low avidity	Hypoechoic	Laparoscopic splenectomy
Raman et al. (16)	37	F	IF	Hypodense mass with lobulated borders<br>CE: Peripheral enhancement on the early phase and heterogeneously enhancement on the late phase				Splenectomy
Raman et al. (16)	67	M	IF	Hypodense mass with smooth border<br>CE: Peripheral enhancement on the early phase and heterogeneously enhancement on the late phase				Splenectomy
Subhawong et al.(28)	27	F	RUQ pain	Hypodense mass	T1WI: Hypointense<br>T2WI: Hyperintense<br>DIW: no restricted diffusion<br>CE: Progressive enhancement with near fill-in by 3 minutes and central areas of hypointensity radiating toward the periphery consistent with scar	Intermediate avidity (SUVmax 2,2)		Splenectomy
Thacker et al.(17)	80	M	IF	Hypodense lesion<br>CE: Peripheral enhancement with delayed central filling	T1WI: Hypointense<br>T2WI: Hypointense<br>CE: Initial intense enhancement of the periphery of the lesion with progressive appearance of thin enhancing septae penetrating the center of the lesion from the periphery	High avidity (SUVmax 4,5)		Splenectomy
Vigorito et al.(18)	56	F	IF	Isodense with smooth border<br>CE: Peripheral enhancement with delayed central filling	T1WI: Isointense<br>T2WI: Hypointense<br>DIW: no restricted diffusion<br>CE :Early phase shows peripheral and septa enhancement and progressive and centripetal filling, with non-enhancing central hypointense scar on delayed phase	Low avidity (SUVmax 4,3)	Hypoechoic	Splenectomy
Wang et al.(2)	29	M	LUQ pain	Hypodense mass				Splenectomy
Watanabe et al.(29)	50	F	IF	Hypodense mass	T1WI: Isointense<br>T2WI:Hypointense		Hypoechoic<br>CE: spoke-wheel-enhancement and persisting peripheral on the delayed phase	Laparoscopic splenectomy

Yoshimaru et al.(30)	57	M	IF	Hypodense mass	T1WI: Isointense<br>T2WI: Heterogeneous isointense<br>DIW: Heterogenous hyperintense and hypointense<br>CE: The early phase demonstrates central hypointensity and the mass is heterogeneously enhanced on the delayed phase image			
Zeeb et al.(59)	36	F	LUQ pain	Hypodense mass				
